# Analysis of Deep Learning-Based Phase Retrieval Algorithm Performance for Quantitative Phase Imaging Microscopy

**DOI:** 10.3390/s22093530

**Published:** 2022-05-06

**Authors:** Sarinporn Visitsattapongse, Kitsada Thadson, Suejit Pechprasarn, Nuntachai Thongpance

**Affiliations:** 1 Department of Biomedical Engineering, School of Engineering, King Mongkut’s Institute of Technology Ladkrabang, Bangkok 10520, Thailand; sarinporn.vi@kmitl.ac.th (S.V.); 62601086@kmitl.ac.th (K.T.); 2 College of Biomedical Engineering, Rangsit University, Pathum Thani 12000, Thailand; suejit.p@rsu.ac.th

**Keywords:** phase retrieval algorithm, quantitative phase imaging, surface plasmon microscopy, instrumentation

## Abstract

Quantitative phase imaging has been of interest to the science and engineering community and has been applied in multiple research fields and applications. Recently, the data-driven approach of artificial intelligence has been utilized in several optical applications, including phase retrieval. However, phase images recovered from artificial intelligence are questionable in their correctness and reliability. Here, we propose a theoretical framework to analyze and quantify the performance of a deep learning-based phase retrieval algorithm for quantitative phase imaging microscopy by comparing recovered phase images to their theoretical phase profile in terms of their correctness. This study has employed both lossless and lossy samples, including uniform plasmonic gold sensors and dielectric layer samples; the plasmonic samples are lossy, whereas the dielectric layers are lossless. The uniform samples enable us to quantify the theoretical phase since they are established and well understood. In addition, a context aggregation network has been employed to demonstrate the phase image regression. Several imaging planes have been simulated serving as input and the label for network training, including a back focal plane image, an image at the image plane, and images when the microscope sample is axially defocused. The back focal plane image plays an essential role in phase retrieval for the plasmonic samples, whereas the dielectric layer requires both image plane and back focal plane information to retrieve the phase profile correctly. Here, we demonstrate that phase images recovered using deep learning can be robust and reliable depending on the sample and the input to the deep learning.

## 1. Introduction

Quantitative phase imaging [1] (QPI) is an imaging technique capturing the optical phase difference of samples due to changes in sample refractive index and optical path length. It is well established that phase imaging can provide a higher sensitivity than intensity imaging due to the phase measurement being more robust to the noise [2]. Another key advantage of QPI is that it is a strong candidate for transparent specimen imaging [3]. QPI has been employed and demonstrated its potential in many applications, including thin-film measurement [4], nanoparticle imaging [5], cell imaging [6], blood screening [1], nanoscale cell structure [7], real-time phase imaging [8], and neuroscience [9]. However, it does have disadvantages, including that it requires an optical interferometer and a well-controlled measurement environment [10], such as vibration isolation and a temperature control system. An optical interferometer is a phase measurement technique based on an interference phenomenon between a reference beam and a signal beam from a sample.

Several well-known QPI techniques include surface plasmon resonance (SPR)-based phase imaging microscopy [11,12,13] and dielectric waveguides [14]. Here, the SPR and the dielectric waveguide have been employed as examples in the analysis. It is essential to point out that the two cases have their unique optical properties; the SPR is a lossy and leaky surface wave [15], whereas the dielectric waveguides are lossless optical samples [16].

There is an interest in performing optical phase imaging with no optical interferometry using a computational phase retrieval algorithm (PR) adopted from X-ray interferometry [17,18]. The PR algorithms work out the phase profile from the wave propagation relationship between the image and Fourier planes. For example, Gerchberg-Saxton [19] uses an iterative approach to calculate the phase profile of a pair of Fourier plane (back focal plane: BFP) and image plane (IMP) images. The transport of intensity equation [20] utilizes several defocused images to solve the phase profile by working out the propagating wave relationship. However, the PR methods, such as Gerchberg-Saxton and ptychography [21] algorithms, still have some disadvantages. Some rely on the iterative approach in the same way as the Gerchberg-Saxton algorithm; meanwhile, some are based on solving finite differences, i.e., the transport of intensity equation, and sometimes the PR cannot provide the correct phase profile as the algorithm reaches one of the local maxima or minima [22].

An alternative approach to PR is to employ deep learning to determine the hidden relationship between intensity images [23,24]. The deep convolutional neural network (DCNN) is a class of deep neural networks (DNN). DCNN is the computational method that mimics the human brain’s neural network to learn and recognize the information. Recently, DCNN has been a popular method for image processing [25,26] and computer vision tasks [27] because of its image pattern recognition capability using the convolutional process. Furthermore, in microscopy and imaging, it was employed to improve microscopy techniques, such as super-resolution [11,28], image denoising [29,30], and image reconstruction [31].

Our previous work experimentally demonstrated that the SPR phase could be retrieved using deep learning with a single BFP image [32], and artificial intelligence (AI) can learn from a synthetic dataset and later be validated using experimental data [33,34]. The AI recognizes the data’s spatial pattern to identify the relationship between the input and output images. The other advantage of using a simulated training dataset is that AI can learn from noiseless data. It has been established that noisy data can obscure and delay the training accuracy and progress; moreover, it can even untrain a trained network. Noise analysis studies for AI-based SPR microscopy and SPR measurement are reported and discussed in our previous publications [32,35].

It is essential to point out that AI can generate high-resolution phase images and enhance features in images. It then comes down to a significant concern of whether the generated image has the correct phase profile. Here, we propose a theoretical framework to compare and quantify the performance of phase retrieval networks trained using simulated images at multiple planes, including the back focal plane, focal plane image, and defocused images.

Furthermore, different combinations of inputs will be discussed as suitable for different samples. The samples included in this study are two types of uniform layer structures: a layer of a uniform plasmonic gold layer deposited on a glass substrate and a uniform dielectric layer coated on a uniform glass substrate. The reasons to employ the uniform samples are that, firstly, their phase profiles can be analytically computed, and they are well known and established so that the discrepancies of a recovered image can be compared against the theoretical phase. The significant difference between the two types of samples is that the plasmonic case is a lossy structure, and the dielectric waveguide is a lossless structure. To the best of the authors’ knowledge, the theoretical framework to evaluate the deep learning-based phase retrieval microscopy’s performance has never been investigated and reported before.

## 2. Materials and Methods

### 2.1. Simulated Microscopic Imaging System

**Figure 1:**
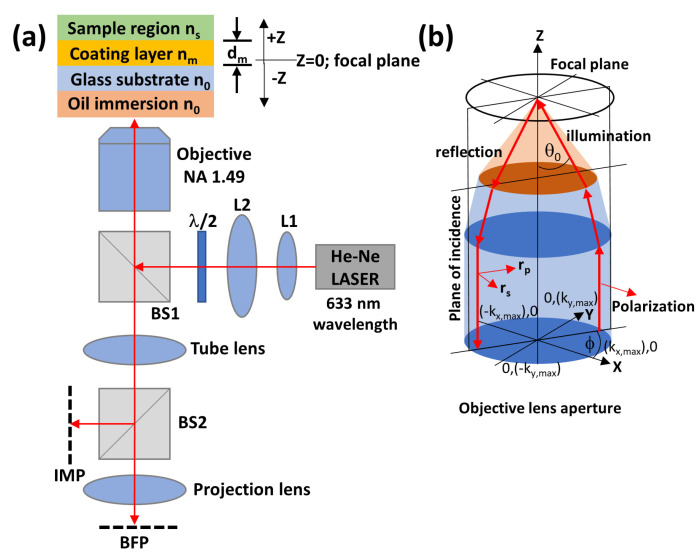
(**a**) Schematic diagram of the microscope system simulated in the study, and (**b**) electric field direction of the linear polarization of the incident wave and the reflected wave.

Figure 1 shows a conventional inverted microscope configuration employed in this study. The system consists of a linearly polarized He-Ne laser at 633 nm wavelength λ, a pair of lenses L1 and L2. The expanded beam passes through a half waveplate and a beam splitter. The split beam is then focused on a sample through an objective lens with an oil immersion numerical aperture (NA) of 1.49. The sample then reflects the light through the objective lens and later forms the focal plane image IMP through the tube lens, and the BFP is imaged through the projection lens, as depicted in Figure 1a. The images at different defocused image planes are captured at the IMP position when the sample is axially defocused or z defocused from the objective lens focal plane. Note that z > 0 means the sample is defocused away from the focal plane and outward the objective lens; z = 0 means the sample is at the focal plane, and z < 0 means the sample is defocused towards the objective lens, as depicted in Figure 1a.

There were two types of samples investigated in this study: (1) uniform SPR samples consisting of a uniform plasmonic gold layer with the layer thickness d_m_ and refractive index n_m_ coated on a standard BK7 coverslip with glass refractive index n_0_ of 1.52, and (2) dielectric waveguides consisting of a uniform dielectric coating with refractive index n_m_ and the layer thickness d_m_ as depicted in Figure 1a.

### 2.2. Back Focal Plane, Image Plane, and Defocused Plane Simulation

**Figure 2:**
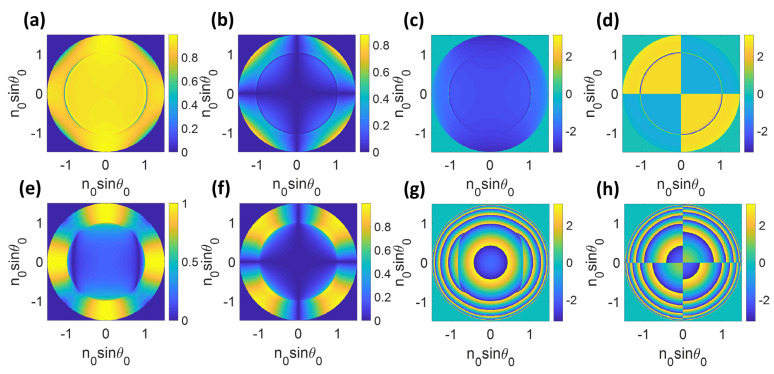
(**a**) |*E_x_*| for 50 nm thick uniform gold sample, (**b**) |*E_y_*| for 50 nm thick uniform gold sensor, (**c**) phase of *E_x_* in (**a**) in rad, (**d**) phase of *E_y_* in (**b**) in rad, (**e**) |*E_x_*| for 1000 nm thick uniform PMMA sample, (**f**) |*E_y_*| for 1000 nm thick uniform PMMA sample, (**g**) phase of *E_x_* in (**e**) in rad, (**h**) phase of *E_y_* in (**f**) in rad.

The objective lens’s BFP was modeled by 512 pixels × 512 pixels providing sufficient sampling of the reflectance spectra for the two types of samples, corresponding to Δsinθ_0_ of 0.0038 for each pixel. Incident plane waves with wave-vectors along the x-axis *k_x_*, the y-axis *k_y_*, and the z-axis *k_z_* represent plane waves at the exit pupil of the objective lens, as shown in Figure 2a for each BFP array. The k_x_ and k_y_ that the objective lens space is in the range of ± $2\pi NA/\lambda_{0}$ and the *k_z_* is given by $\sqrt{{\left(\frac{2\pi n_{0}}{\lambda_{0}}\right)}^{2}-k_{x}^{2}-k_{y}^{2}}$.

The Fresnel equations and the transfer matrix approach were employed to compute the complex reflected electric fields for s-polarization *r_s_* and p-polarization *r_p_* for each incident plane wave θ_0_ corresponding to each array position in the BFP array, as shown in Figure 1b. The complex reflected electric fields for the s-polarization and the p-polarization were then converted to the cartesian coordinate using Equations (1) and (2) for the complex electric fields along the x-axis *E_x_* and the y-axis *E_y_*, respectively. Figure 2a–d show the |*E_x_*|, |*E_y_*|, phase of *E_x_*, and phase of *E_y_* BFP responses for 50 nm uniform gold sample in an air backing environment; whereas Figure 2e–h show the BFP responses for poly (methyl methacrylate) (PMMA) dielectric coating layer of 1000 nm thick under the microscope configuration in Figure 1a.

(1) $$E_{x}=r_{p}\cos\phi \cos\phi +r_{s}\sin\phi \sin\phi ,\;$$

(2) $$E_{y}=r_{p}\cos\phi \sin\phi -r_{s}\cos\phi \sin\phi ,$$

where *ϕ* is the azimuthal angle in the BFP as depicted in Figure 2b and expressed by $tan^{-1}\left(k_{y}/k_{x}\right)$.

**Figure 3:**
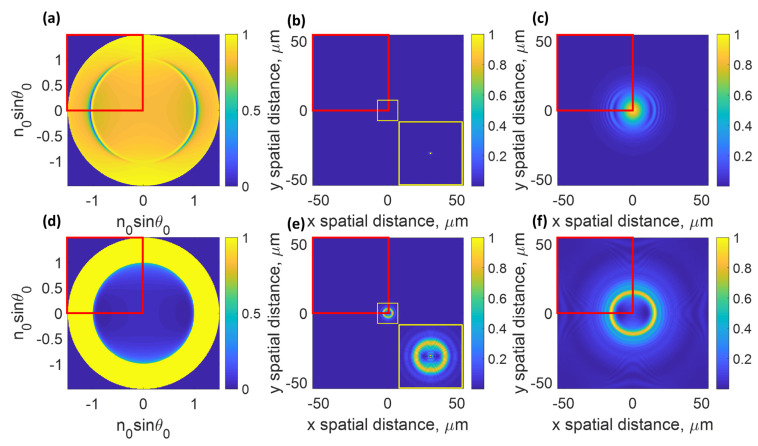
(**a**) BFP image of 50 nm thick uniform gold sample, (**b**) IMP image of 50 nm thick uniform gold sample at z of 0 µm with its zoomed-in image shown in the yellow boxed inset, (**c**) IMP image of 50 nm thick uniform gold sample at z of 6 µm, (**d**) BFP image of 1000 nm thick uniform PMMA sample, (**e**) IMP image of 1000 nm thick uniform PMMA sample at z of 0 µm with its zoomed-in image shown in the yellow boxed inset, and (**f**) IMP image of 1000 nm thick uniform PMMA sample at z of 6 µm.

The BFP image can be computed as expressed in Equation (3) for the intensity image captured at the BFP plane labeled ‘BFP’ in Figure 1a and shown in Figure 3a,d for the SPR case and the dielectric waveguide cases illustrated as examples in Figure 2 and Figure 3.

(3) $$BFP={\left|E_{x}\right|}^{2}+{\left|E_{y}\right|}^{2},$$

Furthermore, the image at different z planes can be computed by taking the inverse Fourier transform of the complex BFP electric fields *E_x_* and *E_y_* in Equations (1) and (2) with the sample defocused phase transfer function as expressed in Equations (4) and (5). The IMP image can be captured at the image plane labeled ‘IMP’ in Figure 1a and calculated using Equation (6) for the intensity image. Figure 3b,e show the IMP images for the example cases when the samples were at the focal plane, whereas Figure 3c,f show the IMP images when the samples were z defocused to 6 µm.

(4) $$IMP_{x}={ℱ}^{-1}\left\{E_{x}e^{2ik_{z}z}\right\},$$

(5) $$IMP_{y}={ℱ}^{-1}\left\{E_{y}e^{2ik_{z}z}\right\},$$

(6) $$IMP={\left|IMP_{x}\right|}^{2}+{\left|IMP_{y}\right|}^{2},$$

### 2.3. Dataset Preparation

#### 2.3.1. Training and Validation Dataset

The training, validation, and test datasets used the BFP and IMP simulation procedure as explained using the parameters shown in Table 1. The training, validation, and test datasets were generated within a range of parameters in Table 1. For SPR samples, the simulated parameters consist of a single layer of a gold thin film with d_m_ of 30 to 60 nm and ±10% of gold refractive index n_m_ from Johnson and Christy 1972 [36] and a sample refractive index n_s_ of 1.0 to 1.4; the incident wavelength λ ranged from 600 nm to 700 nm. The dielectric waveguides were simulated based on the parameters in Table 1. The parameters consist of the dielectric waveguide thickness d_m_ of 0.95 µm to 1.05 µm with the dielectric refractive index n_m_ ranging from 1.20 to 1.50, the sample refractive index ns of 1.00 to 1.40, and the incident wavelength λ of 600 nm to 700 nm. The n_m_ refractive indices covered the typical materials employed in waveguide fabrication and deposition, such as lithium fluoride (LiF) [37], polydimethylsiloxane (PDMS) [38], and PMMA [39]. The linear polarization direction had its electrical field direction pointing on the x-axis. The physical parameters d_m_, n_m_, n_s_, and λ were selected using four uniform random number generators for each parameter producing 4 random physical parameters within its range to simulate 1000 different BFP and IMPs for the training and validation datasets.

There are three types of simulated images, including (1) a BFP intensity calculated using Equation (3), (2) a BFP phase along the x-axis (phase of *E_x_*) calculated by determining the phase of the complex electric fields along the x-axis, in other words, the phase of Equation (1), and (3) IMP intensity images at the focus plane and different z defocus calculated using Equation (6). The difference between the phase of *E_x_* and *E_y_* is that the phase of *E_y_* consists of unsymmetrical phase responses along with the four quadrants, apart from the phase profiles of *E_x_* and *E_y_* are similar, as illustrated in Figure 2. Therefore, the networks for recovering the phase profile of *E_y_* are omitted, as they can be computed from the phase of *E_x_* for the two uniform sample cases. Note that the interface between the glass substrate and the plasmonic gold layer was considered the phase reference point for the Fresnel computations, ensuring no arbitrary phase offset in the phase profiles.

The phase retrieval is performed here in the BFP; however, there is no underlining limitation to applying the proposed method in the other planes, such as the IMP and the defocused IMP. Furthermore, it will be shown in the Section 3 that the angular response in the BFP contains features that can be used to identify optical modes, for example, the SPR [40], Fabry–Pérot [41] and waveguide modes.

In this study, only uniform samples were investigated since the uniform samples allow us to conveniently verify the recovered phase profile compared to the theoretical phase profile simulated using the Fresnel equations and the transfer matrix method explained earlier. Furthermore, the BFP and IMP images contain redundant information in each quadrant due to the twofold symmetry along the x and the y-axes. Therefore, for the DNCC training here, only one quadrant of the images was cropped and employed as the input and the label for the DCNN dataset, as highlighted in the red boxes in Figure 3.

**Figure 4:**
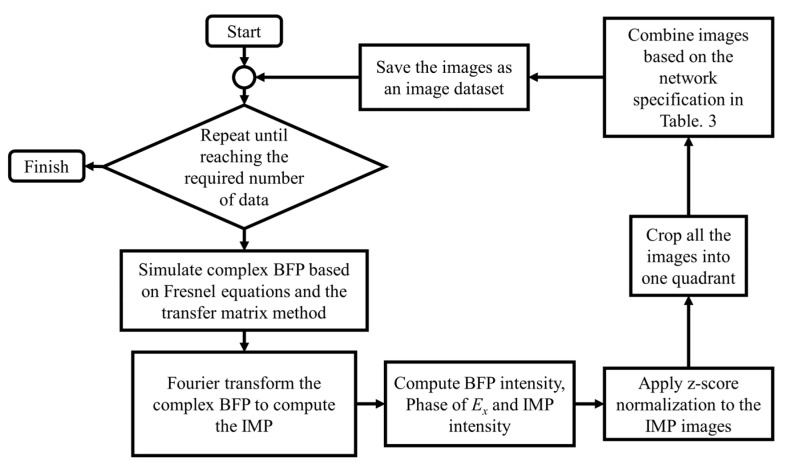
The flowchart of the dataset preparation process.

Figure 4 shows the dataset preparation process. A computed complex BFP was taken to the Fourier transform process for the complex IMP computation, and the z-score normalization was employed to normalize the IMP amplitude. The single quadrant intensity of BFP and IMP images was combined for input data, and the BFP phase profile of E_x_ was employed as the label for supervised training. Each dataset was generated with 1000 sets of input and its corresponding phase label. The dataset was then further separated into 90% and 10% for 900 sets for training and 100 validation datasets, respectively.

#### 2.3.2. Testing Dataset

The cases shown in Table 2 were excluded from the training and validation datasets when used as simulated training and validation datasets for testing purposes, and they were simulated in the same way as discussed in the earlier section.

### 2.4. Context Aggregation Network

Our proposed method is a deep learning-based phase retrieval algorithm. The context aggregation network (CAN) [42] is a CNN architecture for the image-to-image regression task, and it was employed in this research. The network can provide the output in the exact resolution as the input, and it is more adaptable than the other typical CNN by using the adaptive normalizer and the adaptive momentum estimation (ADAM) [43]. Generally, CAN is employed for image processing operators, such as image restoration, enhancement, and denoising [44].

Here, there are five combinations of inputs between BFP and IMP images leading to 5 CAN networks, namely CAN1, CAN2, to CAN5, as shown in Table 3. CAN1 consisted of a single BFP image, recently investigated and experimentally validated by our group [32]. CAN2 was an improved CAN1 by adding a second channel of IMP intensity, equivalent to the input to the Gerchberg-Saxton method. Finally, CAN3, CAN4, and CAN5 were trained with only IMP images at different z defocus planes, equivalent to the input required for the transport of the intensity equation method.

The CAN1 to CAN5 employed the network architecture, as shown in Table 4. The network had ten depth levels. The first level was an image input layer with 256 × 256 × N pixels, where N differed for each CAN network from 1 to 3, as shown in Table 3. Levels 2 to 9 consisted of the convolution layer, the adaptive normalization, and the leaky rectified linear unit (Leaky ReLU or LReLU) activation at a 0.2 scale. In levels 2 to 8, the convolution layers had the dilation and the padding in exponential and extracted the input data to M features; here, the number of required features was also evaluated. It will be shown in the Section 3 later that for the SPR cases, the phase profiles were less complicated than the dielectric waveguide cases. The required network feature M of 64 features can reasonably estimate the SPR phase profile, whereas the dielectric waveguide cases require up to M of 512 due to a more complicated phase response, as shown in Figure 2. At level 8, the receptive field had the exact resolution as the input data. At level 9, the convolutional layer had one dilation and one padding. In the last level, the convolutional layer had the filter size that transforms the data to the exact resolution and channels as the output before sending it to the regression layer.

In this research, the networks were trained in the environment of MATLAB R2019a and NVIDIA Titan RTX single GPU. The training parameters consist of a 0.0001 learning rate, minibatch size of one, and 100 epochs for the dataset, ensuring all the trained networks reached their convergence.

### 2.5. Quantitative Parameter for Performance Evaluation

Here, the recovered phase profiles of the test dataset were compared with their theoretical phase profiles calculated using the Fresnel equations and the transfer matrix approach using structural similarity index measurement (SSIM) [45]. The reason for choosing the wrapped phase profile is to avoid numerical errors due to the unwrapping of phase transitions that were slightly less than 2π rad and phase noise artifacts in the recovered images, which will be shown and discussed in the Section 3.

Note that a constant phase offset is added to the SSIM computation to determine the minimum value of SSIM since the theoretical phase profile and the recovered phase profile can have an arbitrary phase difference. Note that the SSIM value is between 0 and 1, where the SSIM of 0 indicates no similarity between the two images, whereas the SSIM of 1 indicates the highest similarity level.

## 3. Results and Discussion

### 3.1. SPR Samples

#### 3.1.1. CAN1 and CAN2

**Figure 5:**
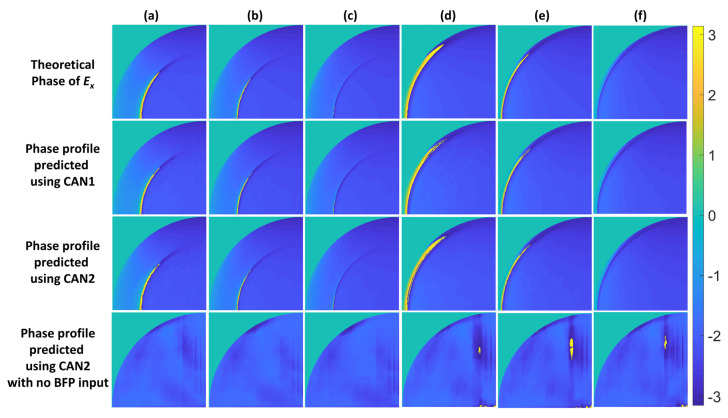
Theoretical phase profiles computed using Fresnel equations and the transfer matrix approach and predicted phase profiles using CAN1, CAN2, and CAN2 with no BFP input for the SPR test data: (**a**) No. 1, (**b**) No. 2, (**c**) No. 3, (**d**) No. 4, (**e**) No. 5, and (**f**) No. 6.

The CAN1 to CAN5 networks were trained with the training and validation SPR dataset with feature number M of 64, as described in Section 2.3. The trained networks were then evaluated using the test dataset to predict the phase responses, as shown in Figure 5. Here, the number of features M was 64, sufficient for the SPR dataset. Table 5 summarizes the SSIM responses calculated for Figure 5. CAN1 and CAN2 can recover the correct phase profiles and provide decent SSIM values for all the SPR test cases. Note that the IMP plane for CAN2 here was at the focal plane z of 0 µm. As a result, CAN1 and CAN2 can recover the SPR phase profile with an average SSIM index of more than 0.90. The performance of the CAN1 and CAN2 had no significant and noticeable difference.

It is interesting to identify what information CAN2 relied on to work out the phase profile. Here, one of the two inputs to CAN2 was switched off to determine the SSIM when the CAN2 network had only one input, as shown in the last two columns of Table 5. The predicted phase profiles of CAN2 with only one BFP still performed well with similar performance to the CAN1; conversely, CAN2 cannot predict a correct phase using a single IMP image, as shown in the bottom row of Figure 5. The results of CAN2 strongly indicate that the CAN2 network mainly relies on the pattern of the BFP image for phase prediction.

#### 3.1.2. CAN3–CAN5

**Figure 6:**
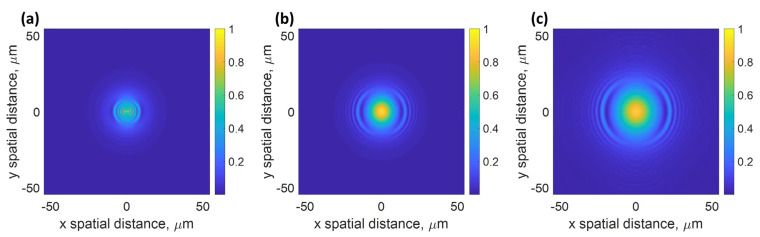
Simulated IMP amplitude at (**a**) −6 µm, (**b**) +6 µm, and (**c**) +9 µm defocus planes for the SPR test data No. 2.

The next question is how the network performs if the network is forced to learn only from the IMP image by giving the CAN3 with no BFP image. Table 6 shows the average SSIM values comparing the theoretical phase profiles of the test cases to the recovered phase profiles for CAN3 to CAN5 at different sample z defocused planes. Note that the SSIM values for all the test cases were similar, and there was no significant performance difference; therefore, the average SSIM values are presented in Table 6. The CAN3 network can recover the phase profiles of the test cases well; however, the performance depends on the z defocus distance. The higher z defocus expands the IMP image, in other words, occupying a larger area in a camera, as depicted in Figure 6.

Figure 6a,b show the simulated IMP images at the z defocused to −6 µm and 6 µm for test data No. 2. It can be seen that the optical intensity profiles are distributed in a larger area compared to when the sample is at the focal plane, shown in Figure 3b. The difference between the two z defocused IMP images is the propagation direction of the surface plasmon polaritons (SPPs). For the negative z defocus, the SPPs propagate inwards, forming a more confined standing wave pattern in the IMP image, as shown in Figure 6a. On the other hand, the SPPs propagate away from the illumination beam, and there is no standing wave pattern observed at the central part of the illumination beam, as depicted in Figure 6b. Note that the standing wave patterns are formed by interference between the SPPs and the other angles, not contributing to the SPP excitation. Therefore, it is more demanding in terms of the number of camera pixels to image the negative defocus pattern due to its confined standing wave pattern around the central part of the IMP. The interference pattern is why the negative defocus can enhance the SSIM compared to the focal plane image. For the positive z defocus, the interference pattern was not as confined as in the negative z defocus, and the interference appeared at the outer part of the image, reducing the demand of the camera pixel. However, when the positive z defocus was too high, the region where the SPPs and the other beam with a decent amplitude decreased, leading to a lower SSIM performance, as depicted in Figure 6c for z of 9 µm. For CAN4, the z defocus distance between 2 IMP planes improved the SSIM performance from 0.7884 for the z defocuses of 6 µm and 7 µm to 0.8348 for the z defocuses of 6 µm and 9 µm. However, when the sample was defocused further to 10 µm, the SSIM performance degraded to 0.7901, indicating that the camera pixels could not accommodate the footprint size of the optical illumination. A similar effect was also found in CAN5. There is a trade-off between the interference pattern contrast and defocused point spread function size in the IMP.

Chow et al. [46] recently demonstrated that the negative z defocused image can be applied to quantitative phase SPR microscopy through the period of the standing wave pattern in the image plane.

**Figure 7:**
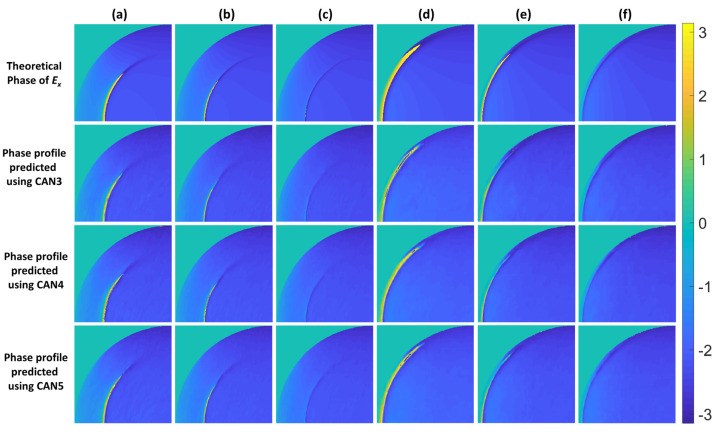
The recovered phase profiles using CAN3, CAN4, and CAN5 for the SPR test cases: (**a**) No. 1, (**b**) No. 2, (**c**) No. 3, (**d**) No. 4, (**e**) No. 5, and (**f**) No. 6.

For this study, the z of 6 µm was chosen for CAN3 to CAN5. The difference between the three networks is the number of IMP planes. For CAN3, there was only a single IMP image, whereas the other two networks, CAN4 and CAN5, required 2 IMP images and 3 IMP images at different defocuses, respectively. Table 6 also shows the average SSIM values of CAN4 and CAN5 for different z defocuses. The best SSIM performance for CAN4 was at the two IMP planes of 6 µm and 9 µm, and for CAN5, the best SSIM was at the three IMP planes of 6 µm, 7.5 µm, and 9 µm, respectively. The SSIM values of CAN3 to CAN5 were 0.8228, 0.8348, and 0.8188, respectively. They were less than the performance of CAN1 and CAN2, as discussed in Table 5 earlier. Figure 7 shows the theoretical phase profiles compared to the recovered phase profiles from CAN3 to CAN5 for all the SPR test data, and Table 7 summarizes the corresponding SSIM calculated from the results in Figure 7. The recovered phase profiles from CAN3 to CAN5 contained more noticeable random noise artifacts than those recovered using CAN1 and CAN2 in Figure 5.

The SSIM values of the three networks show no significant difference. However, CAN4 performed slightly better than CAN3 and CAN5 for all the test data. That means adding the third channel of the input data for CAN5 does not improve the network.

The five types of a trained network can correctly estimate the phase information for the SPR cases in a range of simulated training parameters. For example, the dataset based on the BFP amplitude of CAN1 and CAN2 can approximate the better phase compared to the dataset based on the IMP amplitude of CAN3, CAN4, and CAN5 by 10% in SSIM. For the SPR cases, the BFP image is more crucial for phase prediction than the IMP because the SPR dips with the lossy coupling nature appearing as an apparent dark band in the BFP; it is easier for the CAN to estimate the phase around the BFP intensity dip.

**Figure 8:**
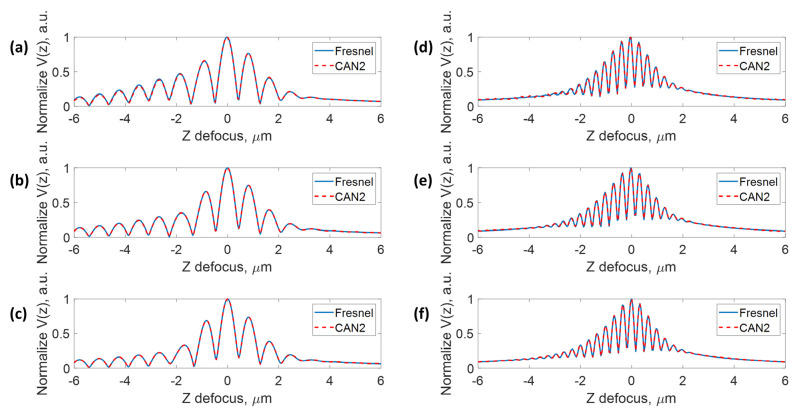
The V(z) signals for the six test datasets comparing the theoretical phase computed using Fresnel equations and the recovered phase profiles from the CAN2 network. The solid blue curves show the V(z) signals computed using the ideal phases calculated using Fresnel equations, and the dashed red curves show the V(z) signals computed using the recovered phases from CAN2 for (**a**) test data No. 1, (**b**) test data No. 2, (**c**) test data No. 3, (**d**) test data No. 4, (**e**) test data No. 5, and (**f**) test data No. 6.

The next question is whether the quality of the recovered BFP phase profiles can represent a practical phase measurement. We adopted confocal surface plasmon V(z) microscopy [47,48] to measure the relative phase between the surface plasmons and a reference beam as the plasmonic sample is defocused towards the objective lens. Figure 8 shows the six test datasets’ V(z) signals. The solid blue curves show the V(z) signals computed using the ideal phases calculated using Fresnel equations, and the dashed red curves show the V(z) signals computed using the recovered phases from CAN2 in Figure 7. The proposed deep learning phase retrieval method can recover phase patterns that provide a similar measurement performance to their theoretical phase.

In the next section, the lossless dielectric waveguide samples are discussed. The advantage of the BFP intensity is no longer valid for the dielectric waveguide samples since there is no pronounced BFP intensity dip, as shown and discussed in Figure 3d earlier.

### 3.2. Dielectric Waveguide Lossless Structures

#### CAN1, CAN2, and CAN4

Another set of CAN1, CAN2, and CAN4 networks was then trained using the training and validation dataset for the dielectric waveguides using the feature number M of 64 for 100 epochs, as listed in Table 1. CAN1 performed the best in the SPR case, CAN2 was also employed here to evaluate the importance of BFP and IMP contributing to the phase retrieval, and CAN4 was the best performance network requiring two IMP images.

Table 8 shows the SSIM values comparing the dielectric waveguide test cases’ recovered phase to their theoretical phase profile. Again, CAN2 performed better than CAN1 and CAN4 by 7% and 6%, respectively. As in the SPR cases, each input to the CAN2 was switched off internally. CAN2 with no BFP input and no IMP input had the SSIM values of 0.2805 and 0.3127, respectively, indicating that the CAN2 network relied on the two planes for estimating the BFP phase information.

It is essential to point out that phase profiles for the dielectric waveguides are more complicated than the SPR cases, and the M of 64 is not sufficient to encapsulate all the BFP phase features. The critical parameter for a more complex sample is how clever the network is. For the SPR, it consisted of only one SPR dip; it, therefore, only required a simpler network. The dielectric waveguide samples consisted of multiple guided modes. It, essentially, required a more sophisticated network architecture, i.e., deeper hidden layers or higher learnable variables. Table 9 shows the average SSIM values for CAN1, CAN2, and CAN4 trained for 100 epochs for the number of features M of 64, 128, 256, and 512. The M of 512 was the memory limit of the GPU employed in this study. Moreover, the higher M required a longer training time. For example, for CAN2 with the M of 64, it took 12 h to train, in contrast to CAN2 with the M of 512, which took around 50 h.

Although the proposed deep learning-based method requires a substantial amount of time for the network training, once it is trained, it can be readily employed for rapid and real-time phase retrieval for the trained measurement system, such as the presented optical microscope system. On the other hand, iterative computation [49] and finite element-based approaches require a lengthy computational time and resource for every recovered image [50]. In general, the AI-based approach is more feasible for real-time measurement applications. Several authors have recently reported artificial intelligence-based real-time microscopic imaging applications [51,52].

**Figure 9:**
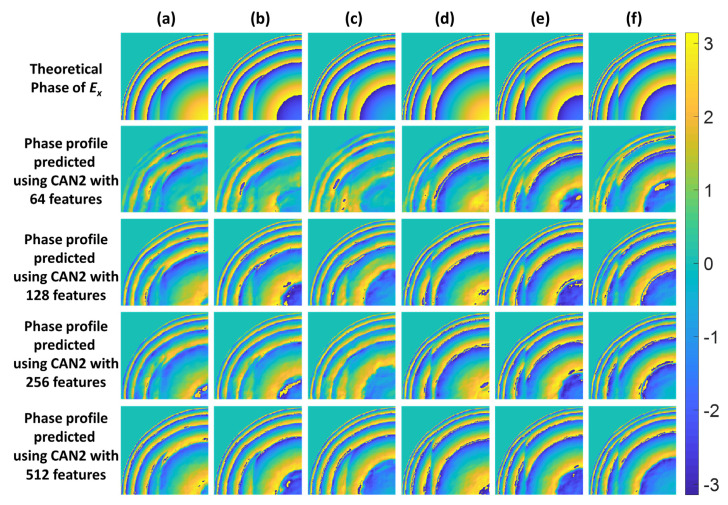
The recovered phase profiles using CAN2 with M of 64, 128, 256, and 512 for the dielectric waveguide test cases: (**a**) No. 1, (**b**) No. 2, (**c**) No. 3, (**d**) No. 4, (**e**) No. 5, and (**f**) No. 6.

Nevertheless, as in Table 8, CAN2 with M of 512 had the SSIM of 0.7406, outperforming the other two networks by 9% and 8% compared to CAN1 and CAN4, respectively. Figure 9 shows the recovered phase profiles of the test cases using CAN2 trained with the different M values compared to their theoretical phase profile. It can be seen that the phase profiles become less noisy and show sharper edge responses at the 2π rad phase wrapping positions for the higher M values.

The CAN requires both the BFP and the IMP to provide a decent phase retrieval for the dielectric waveguides. Therefore, it can be concluded that both planes are essential for phase retrieval. However, the BFP alone cannot predict a correct phase response for the dielectric waveguides since there was no noticeable intensity pattern beyond the critical angle to determine the phase; meanwhile, the IMP images can recover the correct phase profile with the expense of the noise performance compared to the combination of the IMP and BFP planes.

The proposed method can recover the correct phase patterns of the two sample types; however, random phase noise artifacts in the recovered images degrade the overall SSIM performance. Meanwhile, the proposed deep learning-based phase retrieval algorithm did not predict a persisting incorrect pattern or a noticeable phase deviation for all the test cases. In contrast to the Gerchberg-Saxton and other iterative approaches, it is established that the methods can recover a wrong phase pattern due to local minima and maxima [53].

This research confirms that deep learning can be employed to learn the phase relationship in an image through pattern recognition and between several optical planes working out the wave propagation relationship between optical planes to predict the corresponding phase profile. The suitable network configuration for each type of sample differs due to the nature of the BFP intensity profile, and it can only perform phase retrieval within the range of the training dataset. The trained networks are not generalized for different samples. A generalized network to learn the physics of wave propagations in IMP and BFP planes may be possible; however, it will require a more sophisticated network architecture and a more extended range of sample types.

The AI-based microscopy technique is a strong candidate for many applications, including computational microscopy for super-resolution, depth of field enhancement, multi-modal imaging, real-time object classification, object tracking, and biomedical diagnosis. This research has provided a framework and a basis for understanding the behavior of AI under a conventional microscope.

## 4. Conclusions

Here, we have employed the CAN network architecture to evaluate the performance of quantitative phase imaging microscopy. The theoretical framework for analyzing several CAN networks with different input configurations has been proposed and discussed. The input configurations to the networks covered (1) a single BFP input in CAN1, (2) one BFP image and one IMP image for CAN2, (3) one IMP image for CAN3, (4) two IMP images at different sample z defocused planes, and (5) three IMP images at different sample z defocused planes. Two sample types were investigated: the uniform SPR gold samples and the dielectric waveguides. The underlining reason for choosing uniform samples is that the phase responses of the two cases can be computed using Fresnel equations and the transfer matrix method, and they are well established and understood. The difference between the two types is that the SPR samples are lossy structures; conversely, the dielectric waveguides have no optical energy loss. Therefore, different types of samples are suitable for different input configurations for the network. For the SPR cases, the information in the BFP is dominant compared to the IMP, although the correct phase profile can be retrieved using the IMP alone. However, the SSIM performance of the phase profile recovered using the IMP has 10% greater degradation than the BFP, appearing as more random noise artifacts in the recovered phase profiles.

On the other hand, the dielectric waveguides require BFP and IMP to recover the phase profile correctly. The phase profiles cannot be recovered using the BFP alone since there is no intensity dip in the BFP beyond the critical angle. Although the single IMP image or several IMP images in CAN3 to CAN5 successfully recovered the phase profiles of all the test cases, the SSIM performance was lower than CAN2 by almost 10%. It can also be concluded that deep learning can predict phase profiles and learn the relationship between optical planes.

## Figures and Tables

**Table 1:** The simulated parameters for training and validation datasets.

Parameters	Unit	Min	Max
SPR samples			
Medium thickness, d_m_	nm	30	60
Medium refractive index, n_m_	RIU	−10%	+10%
Sample refractive index, n_s_	RIU	1.00	1.40
Wavelength, λ	nm	600	700
Dielectric waveguide			
Medium thickness, d_m_	µm	0.95	1.05
Medium refractive index, n_m_	RIU	1.20	1.50
Sample refractive index, n_s_	RIU	1.00	1.40
Wavelength, λ	nm	600	700

**Table 2:** The test datasets for the SPR and the dielectric waveguide cases.

Parameters	Unit	Data No.					
		1	2	3	4	5	6
SPR							
Plasmonic metal thickness, d_m_	nm	30	40	50	30	40	50
Plasmonic metal refractive index, n_m_	RIU	Gold	Gold	Gold	Gold	Gold	Gold
Surrounding medium refractive index, n_s_	RIU	Air	Air	Air	Water	Water	Water
Wavelength, λ	nm	633	633	633	633	633	633
Dielectric waveguide							
Dielectric waveguide thickness, d_m_	µm	0.95	1	1.05	0.95	1	1.05
Dielectric waveguide refractive index, n_m_	RIU	PMMA	PMMA	PMMA	PMMA	PMMA	PMMA
Surrounding medium refractive index, n_s_	RIU	Air	Air	Air	Water	Water	Water
Wavelength, λ	nm	633	633	633	633	633	633

**Table 3:** List of networks with dataset information.

No.	Input				Label/Output
	BFP	1st IMP	2nd IMP	3rd IMP	BFP Phase
CAN1	✓				✓
CAN2	✓	✓			✓
CAN3		✓			✓
CAN4		✓	✓		✓
CAN5		✓	✓	✓	✓

**Table 4:** The architecture of CAN with ten depths and extract in M features.

Layer	Activations	Learnable	Descriptions
Image input	256 × 256 × N	-	256 × 256 × N images
Convolutional	256 × 256 × M	Weights 3 × 3 × 1 × M, Bias 1 × 1 × M	1 padding, 1 stride
Adaptive normalization		Offset 1 × 1 × M, Scale 1 × 1 × M	-
Leaky ReLU		-	Scale 0.2
Convolutional		Weights 3 × 3 × M × M, Bias 1 × 1 × M	2 padding, 1 stride, 2 dilation
Adaptive normalization		Offset 1 × 1 × M, Scale 1 × 1 × M	-
Leaky ReLU		-	Scale 0.2
Convolutional		Weights 3 × 3 × M × M, Bias 1 × 1 × M	4 padding, 1 stride, 4 dilation
Adaptive normalization		Offset 1 × 1 × M, Scale 1 × 1 × M	-
Leaky ReLU		-	Scale 0.2
Convolutional		Weights 3 × 3 × M × M, Bias 1 × 1 × M	8 padding, 1 stride, 8 dilation
Adaptive normalization		Offset 1 × 1 × M, Scale 1 × 1 × M	-
Leaky ReLU		-	Scale 0.2
Convolutional		Weights 3 × 3 × M × M, Bias 1 × 1 × M	16 padding, 1 stride, 16 dilation
Adaptive normalization	256 × 256×M	Offset 1 × 1 × M, Scale 1 × 1 × M	-
Leaky ReLU		-	Scale 0.2
Convolutional		Weights 3 × 3 × M × M, Bias 1 × 1 × M	32 padding, 1 stride, 32 dilation
Adaptive normalization		Offset 1 × 1 × M, Scale 1 × 1 × M	-
Leaky ReLU		-	Scale 0.2
Convolutional		Weights 3 × 3 × M × M, Bias 1 × 1 × M	64 padding, 1 stride, 64 dilation
Adaptive normalization		Offset 1 × 1 × M, Scale 1 × 1 × M	-
Leaky ReLU		-	Scale 0.2
Convolutional		Weights 3 × 3 × M × M, Bias 1 × 1 × M	128 padding, 1 stride, 128 dilation
Adaptive normalization		Offset 1 × 1 × M, Scale 1 × 1 × M	-
Leaky ReLU		-	Scale 0.2
Convolutional		Weights 3 × 3 × M × M, Bias 1 × 1 × M	1 padding, 1 stride
Adaptive normalization		Offset 1 × 1 × M, Scale 1 × 1 × M	-
Leaky ReLU		-	Scale 0.01
Convolutional	256 × 256 × 1	Weights 1 × 1 × M, Bias 1 × 1	0 padding, 1 stride
Regression	-	-	Mean square error

**Table 5:** The SSIM values of CAN 1 and CAN 2 phase prediction.

Data No.	CAN1	CAN2	CAN2 (BFP Switched Off)	CAN2 (IMP Switched Off)
1	0.8589	0.8767	0.4760	0.8686
2	0.9149	0.9232	0.5027	0.9274
3	0.9237	0.9293	0.5109	0.9300
4	0.8792	0.8752	0.4321	0.8725
5	0.9330	0.9235	0.4420	0.9227
6	0.9479	0.9379	0.4692	0.9302
Average	0.9096	0.9110	0.4721	0.9085

**Table 6:** The average SSIM values for CAN3, CAN4, and CAN5 trained for 100 epochs and the SPR test dataset.

	CAN3: z Defocus (µm)										
	−15	−12	−9	−6	−3	0	3	6	9	12	15
SSIM	0.8062	0.7991	0.8153	0.7965	0.7559	0.7247	0.7985	0.8228	0.7863	0.7932	0.7457
	**CAN4: z Defocus (µm)**										
	6, 7	6, 8	6, 9	6, 10							
SSIM	0.7884	0.8011	0.8348	0.7901							
	**CAN5: z Defocus (µm)**										
	6, 7, 9	6, 7.5, 9	6, 8, 9								
SSIM	0.8126	0.8188	0.8169								

**Table 7:** SSIM values of CAN3, CAN4, and CAN5 phase prediction.

Data No.	CAN3	CAN4	CAN5
1	0.7928	0.7962	0.7808
2	0.8204	0.8245	0.8121
3	0.8412	0.8414	0.8292
4	0.8291	0.8478	0.8285
5	0.8195	0.8447	0.8282
6	0.8334	0.8543	0.8342
Average	0.8228	0.8348	0.8188

**Table 8:** The SSIM values of the recovered phase profiles using CAN1, CAN2, and CAN4 with M of 64.

Data No.	CAN1	CAN2	CAN2 (BFP Switched Off)	CAN2 (IMP Switched Off)	CAN4
1	0.3843	0.5613	0.2552	0.3499	0.5005
2	0.4797	0.5479	0.2875	0.3556	0.5381
3	0.4387	0.4585	0.2869	0.3037	0.5449
4	0.5753	0.6522	0.2793	0.3112	0.4841
5	0.6162	0.6422	0.2852	0.2954	0.5375
6	0.5517	0.6014	0.2888	0.2603	0.4788
Average	0.5077	0.5772	0.2805	0.3127	0.5140

**Table 9:** The SSIM values of CAN1, CAN2, and CAN4 in various features.

	Number of Features			
	64	128	256	512
CAN1	0.5077	0.6077	0.6234	0.6513
CAN2	0.5772	0.6910	0.7089	0.7406
CAN4	0.5140	0.6153	0.6312	0.6594

## Data Availability

The data presented in this study are available on request from the corresponding author.
